# Differing Within-Household Food Security Statuses Are Associated with Varied Maternal Mental Health Outcomes

**DOI:** 10.3390/nu16101522

**Published:** 2024-05-18

**Authors:** Rachel A. Liebe, Chanit’a Holmes, Sarah A. Misyak

**Affiliations:** 1 Department of Nutritional Sciences, Oklahoma State University, Stillwater, OK 74075, USA; 2 Department of Agricultural and Applied Economics, Virginia Tech, Blacksburg, VA 24061, USA; ccholmes@vt.edu; 3 Department of Human Nutrition, Foods, and Exercise, Virginia Tech, Blacksburg, VA 24061, USA; smisyak@vt.edu

**Keywords:** intrahousehold food security, mental health, maternal health, behavioral food coping, diet quality

## Abstract

Household food insecurity is not necessarily equally experienced by all household members, with mothers often changing their intake first when food resources are limited. The purpose of this study was to understand the association between maternal mental health and intrahousehold differences in food security statuses. A cross-sectional survey was administered to Virginia mothers with low income (August–October 2021), assessing validated measures of food security, mental and physical health and related factors. Participants (*n* = 570) were grouped according to the food security status of adults and children within the household. Linear regression was used to assess the outcomes of interest by group and controlled for key demographic variables. Mothers in households with any food insecurity reported worse overall mental health and used 3–4 more food coping strategies than households experiencing food security (*p* < 0.05). Only mothers in households where adults experienced food insecurity reported significantly greater anxiety and depressive symptoms (61.5 and 58.1, respectively) compared to households experiencing food security (55.7 and 52.4, *p* < 0.001). While any experience of household food insecurity is associated with worse maternal mental health, there were differences by the within-household food security status. Future research should explore screening measures that capture specific household members’ food security to connect households with available resources.

## 1. Introduction

Household food insecurity, defined as limited or inconsistent access to enough food at the household level, inequitably affects caregivers who identify as women (referred to as mothers in this manuscript) with low income [1]. In 2022, 17.3% of all households with children in the United States (U.S.) experienced food insecurity compared to 12.8% of all households [1]. Among households headed by a single mother, the likelihood of food insecurity is even higher (33.1%) [1]. The experience of food insecurity may not be equally felt by all household members [2]. For mothers, the experience of food insecurity may often be further complicated by the responsibility for household food environment management and societal expectations of motherhood [3,4]. Managing the household food environment with limited resources may necessitate using behavioral food coping strategies to stretch limited food resources (e.g., buying cheaper foods, asking family for food or money). When food resources are very limited, mothers often cope by reducing their own intake before limiting the intake of other household members, including men in the household [3,5].

The sense of responsibility for managing the household food environment and the desire to shield other household members from food insecurity may be the result of internalized societal expectations. Intensive mothering describes the pervasive ideology in the US where mothers should devote considerable time, energy and resources to their child(ren) [4]. Within this ideology, a “good” mother will put the needs of their child(ren) above competing interests and prioritize themselves last [4,6]. This belief is particularly pervasive among mothers with low income [6]. When mothers fail to meet high societal standards, such as being unable to shield their child(ren) from the effects of food insecurity, they may experience parental burnout, guilt and poor mental health that impacts their relationship with other household members [7,8,9,10,11].

The existing literature suggests there is a relationship between food security and mental health [3,12,13,14,15,16,17,18]. Although there is limited research on the directionality of this relationship, existing evidence suggests this link is bidirectional [15,19]. For mothers in particular, the experience of food insecurity has been associated with increased perceived stress, anxiety and depressive symptoms, and risk of having a diagnosed mental illness [13,15,20,21]. Increasing severity of food insecurity is associated with worsening mental health outcomes [13,21,22], and using food coping strategies (e.g., reduced quality or quantity of food, reduced other expenses) is a mediator of this relationship [12]. Mothers have reported that relying on food coping strategies can contribute to parental guilt, poor mental health and reduced mental bandwidth [23]. Food insecurity is also associated with poor diet quality and poor physical health [24,25]. Both past physical health and diet quality are positively correlated with mental health [26,27]. The effects of coping with food insecurity may also contribute to poor mental and physical health outcomes among children in the household [9,10,11,28,29].

The existing literature suggests mothers face significant societal pressure to protect their children from experiencing food insecurity and that the effects of food insecurity can impact household members differently [3]. This is supported by the relationship between household food security, maternal mental health and physical health outcomes. Despite this evidence suggesting that a better understanding of intrahousehold food insecurity is needed, there is limited understanding of the impact of differing food security statuses between adults and children within a household on maternal mental health. The purpose of this study is to explore how the food security status of both adults and children in the household is associated with maternal mental health. Understanding this association can be used to identify ways mothers successfully cope with limited resources and identify opportunities for public health practitioners to adapt interventions that address food insecurity to further support coping strategies.

## 2. Methods

A cross-sectional survey was administered electronically to Virginia mothers in households with low income via the electronic survey software Qualtrics (Provo, UT, USA). The survey was available from August to October 2021. While the survey methods have been detailed elsewhere [13], an overview is provided below. Methods were approved by the Virginia Tech Institutional Review Board.

### 2.1. Participants

To be eligible for the survey, respondents needed to identify as an adult woman or non-binary person living in Virginia, have children (under 18 years old) living in their household, speak English and report a household income below the Federal Poverty Level. A pre-test of the survey was administered to fifty participants, and minor modifications were made to the flow of the survey. For this study, participants who reported that adults and children in the household were experiencing food insecurity were excluded because the aim of this research was to explore maternal mental health outcomes in households where adult and child food security status differed.

### 2.2. Measures

The 18-item US Department of Agriculture (USDA) Household Food Security Module was used to assess food security status [30]. This tool has a reference time of 12 months and can be used to assess household, adult and child food security status. An affirmative response is scored as one and a negative response is scored as zero. Scores for adults and children within the household range from 0 to 10 and 0 to 8, respectively, with a higher score suggesting lower food insecurity. For each household, adults and children were categorized as experiencing food security or food insecurity based on the levels outlined by Bickel and colleagues [30]. Participants were categorized into three groups based on adult and child food security status: (i) a reference group where both adults and children in the household are experiencing food security; (ii) Adult-Only Food Insecurity (Adult-Only FI) has adults in the household experiencing food insecurity but the children are food secure; and (iii) Child-Only Food Insecurity (Child-Only FI), where adults who are food secure are living with the children experiencing food insecurity.

Maternal mental health was assessed using five indicators: overall mental health, symptoms of anxiety and depression, perceived stress and life satisfaction. Patient Reported Outcome Measurement Information System (PROMIS) scales were used to assess overall mental health (two-item Global Mental Health 2a Scale v1.2), anxiety symptoms over the previous 7 days (four-item Emotional Distress v1.0—Anxiety—Short Form 4a) and depressive symptoms over the previous 7 days (Emotional Distress v1.0—Depression—Short Form 4a) [31,32,33]. The National Institutes of Health (NIH) Toolbox Perceived Stress adults scale was used to assess perceived stress over the previous 30 days [34]. Scores on PROMIS and NIH scales were standardized to the US population by converting raw scores to T-scores. A T-score of 50 is average, and a 10-point deviation was one standard deviation on all scales in this study. A higher score on all scales indicates a greater presence of that construct (e.g., better mental health, more anxiety symptomatology). Lastly, life satisfaction was measured using the Satisfaction with Life Scale, where scores on the five-item scale range from 5 to 35 [35]. A higher score indicates greater life satisfaction.

Other health and well-being measures include overall physical health and self-reported diet quality, which were evaluated on a Likert-type scale from poor (1) to excellent (5) with single validated questions [36,37]. Social support was assessed using a 10-item modified Duke Social Support Inventory that measured network size and satisfaction [38]. Scores ranged from 10 to 30, with a higher score indicating greater social support. The 15-item Hunger Coping Scales were used to assess three types of behavioral food coping strategies (tradeoffs, financial, and rationing) [39]. A total coping strategies score was developed by summing scores on each subscale and ranged from 0 to 15, with a higher score indicating the usage of more strategies. Demographic data included age, race and ethnicity, assistance program participation, income, people living in the household and educational attainment.

### 2.3. Analysis

Data analysis was conducted using R Version 4.1.2. (R Core Team, Vienna, Austria). Descriptive statistics were calculated to summarize demographic information for each group (Reference, Adult-Only FI, Child-Only FI). Linear regression was used to assess whether mental health, behavioral food coping strategies, physical health, diet quality and social support of mothers from reference households differ from those in Adult-Only FI and Child-Only FI households. The models controlled for key demographic variables, including the number of children in the household, SNAP participation, income, household size and whether the mother reported living with a partner or spouse. Separate models were created to explore differences for mothers who identified as white and Black or African American. The sample size was insufficient to allow for exploration of other racial groups. The statistical significance threshold was set a priori at *p* ≤ 0.05.

## 3. Results

### 3.1. Respondent Characteristics

There were 570 respondents who met the eligibility criteria, of which 55.6% (317) were classified as living in a reference household, 39.5% (225) as Adult-Only FI households and 4.9% (28) were Child-Only FI households. Respondents ranged from 18 to 80 years old and reported living with one to seven children. Children’s age ranged from less than 1 month to 17 years old. Respondents primarily identified as white (63.0%) or Black or African American (25.3%). Reported household income ranged from USD 0 to USD 80,000. Across all groups, 59.1% of respondents reported participating in SNAP. Participants most commonly reported their physical health and diet quality as fair or good. See Table 1 for respondent characteristics by adult/child food security group.

Global mental health ranged from 25.8 to 64.6, with a mean of 44.3 ± 10.1, compared to a U.S. average score of 50.0. Anxiety symptoms (mean: 61.2 ± 9.5) ranged from 40.3 to 81.6, and depressive symptoms (60.3 ± 10.4) ranged from 41 to 79.4. Across all respondents, the mean perceived stress score was 61.3 ± 10.7. Mean life satisfaction was 17.9 ± 7.6. The mean social support score for respondents was 20.2 ± 4.9, and respondents reported using 7.3 ± 3.8 behavioral food coping strategies. Table 2 shows the mental health, behavioral food coping strategies and social support results by adult/child food security group.

### 3.2. Effect of Food Security Group on Outcomes of Interest

Table 3 shows the results of each regression, which consists of food security status, household size, household income, number of children in the household, whether a spouse or partner lived in the household and SNAP participation. Our results indicate that mothers in Adult-Only FI and Child-Only FI households reported consistently poorer mental health and well-being than the reference group. In the global mental health regression, where the significant variables were food security status and SNAP participation, mothers from Adult-Only FI and Child-Only FI households had lower global mental health (*p* < 0.001 and *p* = 0.016, respectively) compared to mothers in reference households. Similarly, mothers from Adult-Only FI and Child-Only FI households had greater anxiety (+5.8 and +2.8, respectively), although the effect was only significant for mothers in Adult-Only FI households (*p* < 0.01). The other significant variable in the anxiety symptoms model was having a spouse or partner living in the household, which was associated with reduced anxiety symptoms (−1.7, *p* = 0.040). There were also more depressive symptoms for mothers in the Adult-Only FI group (5.6, *p* < 0.001). SNAP participation was also associated with more depressive symptoms (2.3, *p* = 0.01). Mothers in both the Child-Only FI and Adult-Only FI households had higher perceived stress (*p* = 0.0014 and *p* < 0.001) and lower life satisfaction (*p* = 0.0013 and *p* < 0.001) than mothers in reference households. Notably, no other variables were significantly associated with the stress level. Other variables in the model that significantly impacted life satisfaction included income, having a spouse or partner in the household and participation in SNAP. However, the effect of income was practically insignificant (−0.00004).

Mothers in the Child-Only FI and Adult-Only FI groups used 3.1 and 4.0, respectively, more behavioral food coping strategies than mothers in reference households (*p* < 0.001). SNAP participation was associated with a small increase in behavioral food coping strategy usage (0.5, *p* < 0.05) and with reduced social support (−1.2, *p* < 0.05). SNAP participation was also associated with worse physical health (−0.3, 0.0028). Mothers in Adult-Only FI and Child-Only FI households had lower social support (1.6 and 1.9, *p* < 0.001 and *p* = 0.036) and worse physical health (−0.5, and −0.4, *p* < 0.001 and *p* < 0.05) compared to mothers from reference households. As expected, Adult-Only FI mothers had lower diet quality scores than mothers in the reference group (*p* < 0.001). No other variables in the model were significantly associated with diet quality.

### 3.3. Differences in Effect of Food Security Group on Outcomes by Race

There were 359 mothers who identified as white in the sample: approximately 52.3% lived in households experiencing food security (reference), 42.9% mothers were in the Adult-Only FI households and 9% were in the Child-Only FI households. Regression outcomes for mothers who identified as white were generally consistent with the trends seen among the full sample. The Adult-Only FI group reported significantly worse scores for all outcomes compared to the reference group (*p* < 0.001, Table 4). Respondents in the Child-Only FI group significantly differed from the reference group among mothers who identified as white with higher perceived stress (+7.8, *p* < 0.05) and the use of more behavioral food coping strategies (+2.7, *p* < 0.001). Respondents in this group also reported lower life satisfaction (−5.8, *p* < 0.001) and lower social support (−2.6, *p* < 0.05) than the reference group. The only other variable that differed in significance compared to the full sample was income, which did not significantly impact life satisfaction in this model for mothers who identified as white.

Of the 144 mothers who identified as Black or African American, the majority were in reference households (66.7%), 27.7% were from Adult-Only FI households and 5.6% from Child-Only FI households. Among respondents who identified as Black, the Adult-Only FI group reported higher symptoms of anxiety (5.0, *p* < 0.05) and depression (5.7, *p* < 0.05) and more behavioral food coping strategies (3.3, *p* < 0.001) than the reference group. Respondents in the Child-Only FI group also reported significantly more behavioral food coping strategies (4.3, *p* < 0.001) and additionally reported perceived stress (8.7, *p* < 0.05) than the reference group. For most outcome measures, excluding social support, physical health and diet quality, the magnitude of differences for both the Child-Only FI and Adult-Only FI groups compared to the reference group was consistent with mothers who identified as white. A larger household size (1.6) was associated with greater life satisfaction among mothers who identified as Black (*p* < 0.05). Income was also statistically, but not practically, significantly associated with social support (0.0001, *p* < 0.001). Unlike with the full sample and mothers who identified as white, SNAP participation was not significantly associated with any outcome among mothers who identified as Black.

## 4. Discussion

The findings of this study suggest that any experience of food insecurity in the household, regardless of whether it is adult or child food insecurity, is associated with worse mental health outcomes for mothers. Based on these findings, mothers are using coping strategies to deal with limited resource availability but experience negative mental and physical health outcomes with those coping strategies.

Both the Child-Only FI and Adult-Only FI groups reported lower global mental health, higher perceived stress and lower life satisfaction compared to the reference group. This is consistent with existing studies, suggesting that living in a household experiencing food insecurity is associated with worse mental health outcomes than living in a household with food security [3,5,12,13,15,21,22,39]. Additionally, both the Child-Only FI and Adult-Only FI groups reported using significantly more behavioral food coping strategies and having worse physical health than mothers in the reference group. The existing literature also suggests that behavioral food coping strategies and poor physical health have been associated with food insecurity and mental health outcomes [5,12,13,40], with behavioral food coping strategies acting as a mediator in the relationship between food security and mental health outcomes [12]. Lastly, mothers in both the Child-Only FI and Adult-Only FI groups reported less social support than the reference group, which is also consistent with the existing literature. Social support is positively associated with both food security and mental health outcomes and may reduce the negative effects of food insecurity on mental health outcomes [13,14,41,42].

Although any food insecurity in the household was associated with worse mental health outcomes, there were differences between the Adult-Only FI and Child-Only FI groups. The Adult-Only FI group reported anxiety and depressive symptoms that were half a standard deviation above the reference group and a full standard deviation above the US average, suggesting significant symptoms that impact daily life. This is consistent with the literature, suggesting that the experience of household food insecurity is associated with greater symptoms of anxiety and depression [3,13,15,19,21,22,43]. However, the anxiety and depressive symptoms among mothers in the Child-Only FI group were not significantly different from mothers in the reference group. While there is limited evidence on the mental health outcomes for adults experiencing food security in food-insecure households, there is evidence that children in households experiencing food insecurity are more likely to show symptoms of anxiety and depression than children in households that are food secure, regardless of the child’s food security status [44]. This suggests that members of the household who are not directly experiencing food insecurity may still experience mental health effects from the food insecurity of other household members. More research is needed to understand how symptoms of anxiety and depression differ depending on who in the household is experiencing food insecurity. Additionally, anxiety and depressive symptoms among mothers in the Child-Only FI group should be further explored with a larger sample size.

Mothers in the Adult-Only FI group reported significantly worse diet quality than the reference and Child-Only FI groups. People experiencing food insecurity report worse diet quality than those experiencing food security, which may occur because mothers often give limited resources to other household members and experience reductions in dietary quality or quantity before other household members [3,45]. This suggests that the experience of food insecurity may be different for mothers than for other household members. A recent study with children living in households experiencing food insecurity may report experiencing more symptoms of food insecurity than reported by their parents [46]. This finding, along with the findings of the present study, suggests that relying only on measures of food insecurity at the household level may conceal differences in experiences between household members that have physical and mental consequences for mothers.

Although there were significant differences by race in the findings, the direction and magnitude of differences between both the Child-Only FI and Adult-Only FI groups compared to the reference group were relatively consistent for mothers who identified as white and those who identified as Black or African American. This suggests that some of the differences in significance may be attributable to a small sample size in some groups, particularly in the Child-Only FI group among mothers who identified as Black. This warrants further exploration in the future to determine if these findings persist with a larger sample size.

Based on the percentage of the full sample that experienced maternal but not child food insecurity, mothers appear to be shielding their children from experiencing food insecurity, which is in line with other research [3,47,48,49,50]. They reported using more behavioral food coping strategies than mothers in reference households. However, the act of shielding children may be impacting their mental and physical health [50]. Practitioners and future research should identify ways to support mothers as they seek to cope with food insecurity. While educational programming to improve coping or mental health may be beneficial, larger system-level changes to improve circumstances so mothers do not have to give up their resources to support children and other household members are also needed. Additionally, future research should identify measures to capture nuances in food security status between household members to explore opportunities to provide support tailored for household members who are most impacted.

There were several limitations of the present study. First, the small sample size in the Child-Only FI group limited statistical power, especially in the analysis by race. Despite this, differences in mental health outcomes between the reference and Child-Only FI groups were still seen and merit further exploration with a larger sample size. Differences by race should be further explored in greater detail as well. Secondly, mothers completed the survey and, therefore, may have underreported food insecurity among children in the household. However, the USDA Household Food Security Module, completed by one adult within the household, has been assessed as having reasonable sensitivity and specificity [30]. Similarly, for households with more than one adult present, the Household Food Security Module does not allow for distinction between household members regarding food security status. Therefore, we were not able to definitively assess whether another adult within the household was experiencing food insecurity. This limitation should be further explored in the future. Thirdly, participants were randomly invited from all eligible Virginia panel members; therefore, the sample is not necessarily generalizable. Furthermore, this was a cross-sectional design, so no conclusions about causality or directionality can be made. Despite these limitations, the findings of this study suggest a need to further explore the impact of household food insecurity on specific household members with a longitudinal design and a larger, generalizable sample in the future.

## 5. Conclusions

In 2022, the White House Conference on Hunger, Nutrition, and Health prioritized addressing nutrition insecurity [51]. Nutrition security enhances the definition of food security by drawing attention to the importance of equity and nutritional value. Although both equity and diet quality are inherent in the definition of food security, they have sometimes been historically overlooked in an effort to ensure an adequate quantity of food [52]. This focus on quantity contributed to existing inequities and produced significant disparities in physical and mental health outcomes, affecting minoritized populations, especially people with low income [52,53]. The White House Conference on Hunger, Nutrition, and Health called for an integration of nutrition and health through the promotion of more effective screening for nutrition insecurity and referral to community food resources, especially among families with young children [51]. The findings of this study suggest that, to achieve this goal, a screening method that can capture the food security status of all household members must be identified to accurately reflect needs within a household. Additionally, because the findings of this study suggest any experience of food insecurity within a household contributes to poor maternal mental health, public health practitioners should be prepared to provide referral services for affordable mental health care to improve nutrition security to more fully integrate nutrition and health.

## Figures and Tables

**Table 1:** Demographic characteristics of survey respondents by adult/child food security status ^1^.

Construct		Reference	Child-Only FI	Adult-Only FI
		Mean + SD or %	Mean + SD or %	Mean + SD or %
		(317)	(28)	(225)
Age		34.3 + 10.7	35.6 + 8.87	33.5 + 9.91
Household Income		25,915 + 17,115	26,735 + 16,708	24,483 + 14,903
Number of Children		2.08 + 1.12	2.04 + 1.20	1.84 + 1.09
Ages of Children		7.37 + 5.27	7.93 + 4.69	6.77 + 4.99
Total People Residing in Household		4.30 + 1.52	4.39 + 1.85	4.11 + 2.30
SNAP ^2^ Participation (%)		57.41	60.71	61.33
Spouse or Unmarried Partner in Household (%)		65.62	57.14	62.67
Race	White (%)	59.31	60.71	68.44
	Black or African American (%)	30.28	28.57	17.78
	Asian (%)	3.15	10.71	2.22
	American Indian or Alaskan Native (%)	0.00	0.00	0.89
	Two or more races (%)	4.42	0.00	8.44
	Some other race (%)	2.84	0.00	2.22
Ethnicity	Hispanic or Latino (%)	8.52	0.00	9.78
General Health	Poor (%)	3.15	0.00	6.22
	Fair (%)	15.46	39.29	32.44
	Good (%)	39.75	39.29	35.56
	Very Good (%)	26.18	10.71	20.44
	Excellent (%)	15.46	10.71	5.33
Diet Quality	Poor (%)	8.83	10.71	13.33
	Fair (%)	25.87	25.00	39.11
	Good (%)	38.49	50.00	33.33
	Very Good (%)	16.40	10.71	11.56
	Excellent (%)	10.41	3.57	2.67

^1^. Reference: households where adults and children are food secure. Child-Only FI: Households where adults are food secure and children are experiencing food insecurity. Adult-Only FI: Households where adults are experiencing food insecurity and children are food secure. ^2^. SNAP: Supplemental Nutrition Assistance Program.

**Table 2:** Coping strategies, mental health and social support of survey respondents by adult/child food security status ^1^.

Construct	Reference	Child-Only FI	Adult-Only FI
	Mean ± SD	Mean ± SD	Mean ± SD
	(317)	(28)	(225)
Behavioral Food Coping Strategies ^2^			
Tradeoffs	1.29 ± 0.65	1.93 ± 0.96	1.93 ± 0.85
Financial	1.28 ± 1.25	2.43 ± 1.07	3.01 ± 1.43
Rationing	0.96 ± 1.28	2.29 ± 1.63	2.57 ± 1.49
Total	3.53 ± 2.49	6.64 ± 2.51	7.51 ± 2.93
Mental Health ^3^			
Global Mental Health	48.4 ± 9.86	43.8 ± 8.77	42.9 ± 8.95
Anxiety Symptoms	55.7 ± 9.74	58.7 ± 7.48	61.6 ± 8.54
Depressive Symptoms	54.5 ± 9.91	58.0 ± 7.89	60.1 ± 9.86
Life Satisfaction	21.3 ± 7.49	16.5 ± 6.22	17.7 ± 6.90
Perceived Stress	55.7 ± 11.3	62.6 ± 7.86	61.5 ± 10.0
Social Support ^4^			
Network	8.32 ± 2.31	7.68 ± 2.20	7.92 ± 2.18
Satisfaction	13.56 ± 3.36	12.25 ± 2.73	12.40 ± 3.31
Total	21.89 ± 4.77	19.93 ± 4.14	20.32 ± 4.71

^1^ Reference: households where adults and children are food secure. Child-Only FI: Households where adults are food secure and children are experiencing food insecurity. Adult-Only FI: Households where adults are experiencing food insecurity and children are food secure. ^2^ Each scale was out of 5, with a higher score indicating greater usage of coping strategies. ^3^ Scales are standardized to US population with average score of 50. A score above 50 indicated great presence of the construct and a score below 50 indicates less. Ten points is 1 SD. Life satisfaction is a measured on a scale from 5–35, with a higher score indicating greater life satisfaction. ^4^ Social support: There are two subscales: network (out of 12) and satisfaction (out of 18). Total social support is out of 30, with a higher score indicating greater social support.

**Table 3:** Regressions of outcome variables on food security group, household size, income, number of children, spouse and SNAP participation (*n* = 570) ^1^.

	Global Mental Health ^2^	Anxiety Symptoms ^2^	Depressive Symptoms ^2^	Perceived Stress ^2^	Life Satisfaction ^2^	Behavioral Food Coping Strategies ^3^	Social Support ^4^	Physical Health ^5^	Diet Quality ^5^
Intercept	46.61 **	55.7 **	52.54 **	54.91 **	20.98 **	3.6 **	22.44 **	3.52 **	3.03 **
Child-Only FI ^6^	−4.46 *	2.81	3.4	6.77 **	−4.44 *	3.11 **	−1.94 *	−0.41 *	−0.22
Adult-Only FI ^6^	−5.24 **	5.80 **	5.59 **	5.67 **	−3.44 **	3.97 **	−1.55 **	−0.48 **	−0.41 **
Household Size	0.33	−0.12	−0.02	−0.09	0.25	−0.17 *	0.05	0.01	−0.02
Income	0.00	0.00	0.00	0.00	−0.00004 *	0.00	0.00	0.00	0.00
Number of Children	0.58	−0.07	−0.32	−0.15	−0.08	−0.15	−0.31	−0.01	−0.06
Spouse (Yes)	0.94	−1.74 *	−0.59	−0.35	2.91 **	−0.07	−0.13	−0.03	−0.05
SNAP (Yes) ^7^	−1.83 *	1.30	2.28 *	1.78	−2.45 **	0.48 *	−1.19 *	−0.27 *	−0.13

^1^ * denotes *p* < 0.05, ** denotes *p* < 0.001. ^2^ Each scale was out of 5, with a higher score indicating greater usage of coping strategies. ^3^ Scales are standardized to US population with average score of 50. A score above 50 indicated great presence of the construct and a score below 50 indicates less. Ten points is 1 SD. Life satisfaction is a measured on a scale from 5–35, with a higher score indicating greater life satisfaction. ^4^ Social support: There are two subscales: network (out of 12) and satisfaction (out of 18). Total social support is out of 30, with a higher score indicating greater social support. ^5^ Physical health and diet quality were both assessed on a Likert-type scale with 1 being poor and 5 being excellent. ^6^ Reference: households where adults and children are food secure. Child-Only FI: Households where adults are food secure and children are experiencing food insecurity. Adult-Only FI: Households where adults are experiencing food insecurity and children are food secure. ^7^ SNAP: Supplemental Nutrition Assistance Program.

**Table 4:** Regressions of outcome variables on food security group, household size, income, number of children, spouse and SNAP participation by race ^1^.

	Global Mental Health ^2^	Anxiety Symptoms ^2^	Depressive Symptoms ^2^	Perceived Stress ^2^	Life Satisfaction ^2^	Behavioral Food Coping Strategies ^3^	Social Support ^4^	Physical Health ^5^	Diet Quality ^5^
Mothers Who Identified as White (*n* = 359)									
Intercept	45.61 **	56.1 **	53.42 *	54.87 **	21.77 **	3.07 **	22.95 **	3.44 **	2.84 **
Child-Only FI ^6^	−4.30	3.63	3.82	7.79 *	−5.78 **	2.72 **	−2.61 *	−0.48	−0.05
Adult-Only FI ^6^	−5.44 **	5.50 **	4.99 **	5.70 **	−3.58 **	4.26 **	−1.92 **	−0.53 **	−0.47 **
Household Size	0.29	−0.03	−0.08	−0.04	0.06	−0.16 *	0.06	0.01	−0.01
Income	0.00	0.00	0.00	0.00	0.00	0.00	0.00	0.00	0.00
Number of Children	0.45	0.12	0.32	0.04	0.02	0.07	−0.01	0.04	0.06
Spouse (Yes)	2.09	−2.30 *	−1.65	−0.36	3.15 **	−0.04	−0.17	0.07	0.03
SNAP (Yes) ^7^	−1.78	0.89	2.66 *	2.28	−3.13 **	0.97 *	−1.62 *	−0.30 *	−0.15
Mothers Who Identified as Black or African American (*n* = 144)									
Intercept	44.4 **	59.6 **	54.51 *	60.04 **	15.91 **	5.36 **	20.07 **	3.49 **	3.14 **
Child-Only FI ^6^	−5.12	3.39	3.83	8.68 *	−4.06	4.27 **	0.25	0.07	−0.78
Adult-Only FI ^6^	−2.82	5.01 *	5.65 *	4.15	−8.72	3.29 **	0.85	−0.05	−0.17
Household Size	1.31	−1.41	−0.89	−1.54	1.55 *	−0.28	0.24	0.00	0.01
Income	0.00	0.00	0.00	0.00	0.00	0.00	0.0001 **	0.00	0.00
Number of Children	−0.05	0.43	0.92	0.25	−0.94	0.17	−0.75	−0.03	0.00
Spouse (Yes)	0.82	−0.96	0.42	1.55	1.32	−0.11	−0.38	0.09	0.04
SNAP (Yes) ^7^	−2.44	1.49	2.15	0.99	−0.75	−0.41	−0.57	−0.18	−0.03

^1^ * denotes *p* < 0.05, ** denotes *p* < 0.001. ^2^ Each scale was out of 5, with a higher score indicating greater usage of coping strategies. ^3^ Scales are standardized to US population with average score of 50. A score above 50 indicated great presence of the construct and a score below 50 indicates less. Ten points is 1 SD. Life satisfaction is a measured on a scale from 5–35, with a higher score indicating greater life satisfaction. ^4^ Social support: There are two subscales: network (out of 12) and satisfaction (out of 18). Total social support is out of 30, with a higher score indicating greater social support. ^5^ Physical health and diet quality were both assessed on a Likert-type scale with 1 being poor and 5 being excellent. ^6^ Reference: households where adults and children are food secure. Child-Only FI: Households where adults are food secure and children are experiencing food insecurity. Adult-Only FI: Households where adults are experiencing food insecurity and children are food secure. ^7^ SNAP: Supplemental Nutrition Assistance Program.

## Data Availability

The data presented in this study are available on request from the corresponding author. The data are not publicly available due to IRB requirements.
